# Multi-marker testing for cancer: what can we learn from modern prenatal testing for Trisomy-21

**Published:** 2007-02-09

**Authors:** Erasmus Schneider, Gerald Mizejewski

**Affiliations:** Wadsworth Center, New York State Department of Health, Empire State Plaza, Albany, NY 12201

A recent report in the New England Journal of Medicine on prenatal screening for Down’s syndrome (Malone et al 2005) received much attention in the non-scientific press, such as Time (Wallis 2005) and Forbes (Gordon 2005) magazines. This paper describes the results from a multicenter study that showed that, when an integrated, multi-marker approach was combined with a specific algorithm, it was possible to detect 90–95% of fetuses affected with trisomy 21 relatively early in a pregnancy with high accuracy, giving the prospective parents time to make appropriate choices. This study represents the culmination of over 25 years of research that has gradually increased the utility of prenatal screening for chromosomal defects. This effort started in the 1970s with the observation that there was an increased risk for chromosomal abnormalities, primarily trisomy 21, in mothers of advanced age at the time of pregnancy (Hook 1976a, b). In the mid 1980s it was shown that combining age with the results from AFP (alpha-fetoprotein) and beta-hCG (human chorionic gonadotropin) measurements in the maternal serum could increase the detection rate from below 40% to around 50–55% (Bogart et al 1987; Cuckle et al 1987). By 1990, the so-called triple test was developed, which takes into account the maternal age in combination with the levels of AFP, beta-hCG, and uE3 (unconjugated estriol) in the maternal serum during the second trimester of pregnancy, thereby achieving a detection rate of up to 70% for trisomy 21 (Cuckle et al 1988; Wald et al 1989). This detection rate was further improved a few years later with the addition of dimeric inhibin A measurement to form the quadruple test, which increased the detection rate to almost 80% (Wald et al 1994a; Aitken et al 1996). Today, either the triple or quadruple test is the standard of prenatal care for expectant mothers and their unborn child. At the same time that the quadruple test was introduced, various groups started to develop tests that could be used earlier in a pregnancy, i.e, during the first trimester, which led to the combination of the levels for the biochemical serum markers PAPP-A (pregnancy-associated plasma protein A) and free beta-hCG with the physical marker of nuchal translucency determined by ultrasound (Wald et al 1994b; Wald et al 1995; Wald and Hackshaw 1997). These developments finally culminated in two landmark studies, one in England (SURUSS) (Wald et al 2003), the other in the USA (FASTER) (Dugoff et al 2004; Malone et al 2005), that demonstrated that a combination or integration of second trimester screening with first trimester screening gives the highest sensitivity for the prenatal detection of chromosomal defects, while at the same time keeping the false-positive rate low.

So, is there a lesson in this story for the early detection of cancer by screening? We believe so. It clearly shows that a combination of multiple markers, each of which by itself has limited sensitivity and/or specificity, can lead to a powerful screening test. In cancer screening, the traditional approach has been to seek the one and only “silver bullet” type biomarker that detects all cancers early without too many false positive results. The latter characteristic is especially important because of the relatively low incidence of individual cancers among the general population, and the potentially multiple invasive, complex and expensive follow-up tests triggered by a positive screening result. Unfortunately, this magic marker has proven elusive, the occasional claim to the contrary notwithstanding. In contrast, the scientific literature is full of reports on initially promising new bio-markers that, when further investigated, turn out not to be that good after all. But are they really not good, or is it just a matter of the paradigm that they are expected to fit? Perhaps, if a similar strategy to that successfully used in prenatal screening were adopted, some of these markers could be useful after all (Li et al 2005). Why not develop a multi-marker screening approach for cancer? Indeed, some form of multi-marker testing is al-ready in usage, and more are in development. For example, in the USA screening for prostate cancer in men over 50 years of age now consists of a digital rectal exam (DRE; a physical marker) and a PSA (prostate-specific antigen) test in serum (a biochemical marker), that together have a substantially higher detection rate than does either marker alone (Catalona et al 1994; Schröder et al 1998; Schröder et al 2001; Vis et al 2001). Furthermore, in cases where the results put the patient into the elevated risk, yet diagnostically ambiguous grey area – a PSA value between 4 and 10 ng/ml and a negative DRE -a second biochemical marker, namely free PSA, can be added, that helps to distinguish benign from malignant conditions (Catalona et al 1995; Raaijmakers et al 2004). While far from perfect, and while certainly not yet as sophisticated as the integrated screening for fetal defects, this triple-marker approach for prostate cancer has proven capable of detecting a substantial number of early cancers, although the effect of early detection on overall mortality from prostate cancer is still unclear (Postma and Schröder 2005). Other multi-marker test protocols, the ProstAsure index (Babaian et al 1998) and ProstataClass (Stephan et al 2002a, b), use neural network-derived, non-linear algorithms to calculate the risk for prostate cancer from values for three serum markers and the patient’s age (ProstAsure), or free and total PSA, DRE, patient age and prostate volume (ProstataClass). The latter algorithm introduces another physical marker, prostate volume, that is derived by an imaging technique, namely transrectal ultrasound or TRUS (http://www.charite.de/ch/uro/de/html/prostatabiopsie/prostata_en.html, accessed 2/13/2006). In direct comparisons these indices were shown to perform better than percent free PSA alone, and to significantly reduce the number of unnecessary biopsies. However, it is not yet clear whether those early encouraging results will hold up in larger, prospective studies. Several other combinations of markers and algorithms have been proposed (Kamoi and Babaian 1999; Bauer and Moul 2000); it will be interesting to see which, if any, of these will survive more rigorous clinical studies and whether they ultimately will have an impact on prostate cancer mortality.

Another cancer for which a multi-marker approach has been attempted is ovarian cancer, for which currently no single good early detection marker exists. The most widely used biochemical marker for ovarian cancer is CA125 (Bast et al 1998). However, while most advanced-stage cancers show elevated CA125 serum levels, the marker’s sensitivity for early-stage disease is less than 50% (Skates et al 2004). In order to improve early detection sensitivity without sacrificing specificity, one or more additional markers, including, among others, CA72-4, CA15-3, OVX1, lipid-associated sialic acid (LASA,) and macrophage colony-stimulating factor (M-CSF), were added (Terry et al 2004; Bast et al 2005). The various combinations of markers were then evaluated with a number of prediction models and methods, including artificial neural networks, logistic regression, classification trees and mixture discriminant analysis (Berek and Bast 1995; Woolas et al 1995; Zhang et al 2000; Skates et al 2004). As for prostate cancer detection, there was a clear performance improvement when using multi-marker analyses, compared to measurement of CA125 alone. Furthermore, a combination of new bio-markers identified through proteomics strategies also exhibited increased sensitivity and specificity for the early detection of ovarian cancer (Zhang et al 2004). Finally, a novel recursive approach has shown that, instead of measuring multiple individual analytes, it is feasible to analyze many markers simultaneously, using mass spectroscopy to determine patterns of markers, whereby the pattern itself becomes the analytical entity. Thus, the pattern is what is diagnostic for the presence or absence of cancer (Petricoin et al 2002). Although the initial study has generated considerable controversy, intensive efforts are now underway to further develop and validate this strategy.

Together, these examples clearly show that multi-marker screening can have its place in early cancer detection. So what, if anything, is holding us back? *A priori,* there seem to exist no scientific reasons why such an approach should not work for cancer screening. However, a number of issues need to be addressed before any multi-marker test can become the standard of care. As for any new clinical procedure, such a test has to be validated in large, well designed prospective trials.

One of the difficulties is that cancer is a rather complex disease, and therefore the confirmation of whether cancer is indeed present or not after a positive screening result is rarely as easy and unequivocal as a karyotype for trisomy 21. Furthermore, whether the cancer will develop and cause clinical symptoms may not be known for many years, whereas after fetal defect marker testing the ultimate outcome will be known within several months of the analysis. In addition, cancer screening is not just a one-time procedure, in contrast to screening for fetal defects, which has to be conducted only once during a pregnancy. Rather, screening for cancer needs to be periodically repeated during a person’s life, thereby raising a number of other issues. These include cost/benefit and the appropriate interval between tests repetitions.

Finally, what is unique about the triple and quadruple test for prenatal fetal defect screening is that neither uses complex models; from an informatics standpoint, their implementation amounts to simply *counting positively ruling-in markers.* Much of the modeling that is done today in the discovery phase of cancer biomarker studies explores highly computationally intensive multivariate prediction models (logistic regression, CART, PCA, support vector machines, neural networks) using dozens to hundreds, if not thousands of individual markers. We wonder if, and how, these types of prediction models will generalize for use in the clinic, for several reasons. First, there is the technical challenge to accurately and reproducibly measure a large number of markers. Second, the current models seek and use all markers in a panel in all patients in a fixed manner. Third, the output, while resulting in a specific class prediction, can be difficult for clinicians to interpret. This is in contrast to the triple/quadruple test, in which markers that ‘miss’ a positive clinical diagnosis for Trisomy-21 are provided ‘back-up’ by other markers that provide evidence for a positive diagnosis. Finally, the relative performance of models generated using the various alternatives have not been sufficiently explored.

While we think that complex fixed-marker models should continue to be explored, simpler models may be possible and ultimately needed to avoid some of the real and/or perceived limitations that lead to resistance in the adoption of the complex models. Researchers involved in cancer biomarker model development should become aware of and consider simple current clinical diagnostics solutions, such as the triple/quadruple test, as a guide for their own diagnostic model formulation.

These and other key issues obviously need to be addressed before any new test will have a significant impact on early cancer detection. Unless new and better individual cancer-specific markers are discovered, a multi-marker testing approach may hold the greatest promise for the improved early detection of cancer.

## References

[b1-cin-02-44] Aitken DA, Wallace EM, Crossley JA (1996). “Dimeric inhibin A as a marker for Down’s syndrome in early pregnancy”. N Engl J Med.

[b2-cin-02-44] Babaian RJ, Fritsche HA, Zhang Z (1998). “Evaluation of prostAsure index in the detection of prostate cancer: a preliminary report”. Urology.

[b3-cin-02-44] Bast RC, Badgwell D, Lu Z (2005). “New tumor markers: CA125 and beyond”. Int J Gynecol Cancer.

[b4-cin-02-44] Bast RC, Xu FJ, Yu YH (1998). “CA 125: the past and the future”. Int J Biol Markers.

[b5-cin-02-44] Bauer JJ, Moul JW, Resnick MI, Thompson IM (2000). Assessment of risk of prostate cancer: algorithms for diagnosis, staging, and prognosis Advanced therapy of prostate disease.

[b6-cin-02-44] Berek JS, Bast RC (1995). “Ovarian cancer screening. The use of serial complementary tumor markers to improve sensitivity and specificity for early detection”. Cancer.

[b7-cin-02-44] Bogart MH, Pandian MR, Jones OW (1987). “Abnormal maternal serum chorionic gonadotropin levels in pregnancies with fetal chromosome abnormalities”. Prenat Diagn.

[b8-cin-02-44] Catalona WJ, Richie JP, Ahmann FR (1994). “Comparision of digital rectal examination and serum prostate specific antigen in the early detection of prostate cancer: results of a multicenter clinical trial of 6,630 men.”. J Urol.

[b9-cin-02-44] Catalona WJ, Smith DS, Wolfert RL (1995). “Evaluation of percentage of free serum prostate-specific antigen to improve specificity of prostate cancer screening”. JAMA.

[b10-cin-02-44] Cuckle HS, Wald NJ, Barkai G (1988). “First-trimester biochemical screening for Down syndrome”. Lancet.

[b11-cin-02-44] Cuckle HS, Wald NJ, Thompson SG (1987). “Estimating a woman’s risk of having a pregnancy associated with Down’s syndrome using her age and serum alpha-fetoprotein level”. Br J Obstet Gynaecol.

[b12-cin-02-44] Dugoff L, Hobbins JC, Malone FD (2004). “First-trimester maternal serum PAPP-A and free-beta subunit human chorionic gonadotropin concentrations and nuchal translucency are associated with obstetric complications: a population-based screening study (the FASTER Trial)”. Am J Obstet Gynecol.

[b13-cin-02-44] Gordon S 2005 “Test Detects Down Syndrome Early.”Forbes Retrieved Dec. 15, 2005, from http://www.forbes.com/lifestyle/health/feeds/hscout/2005/11/09/hscout529032.html

[b14-cin-02-44] Hook EB (1976a). “Estimates of maternal age-specific risks of Down-syndrome birth in women aged 34–41”. Lancet.

[b15-cin-02-44] Hook EB (1976b). “Risk of Down syndrome in relation to maternal age”. Lancet.

[b16-cin-02-44] Kamoi K, Babaian RJ (1999). “Advances in the application of prostate-specific antigen in the detection of early-stage prostate cancer”. Semin Oncol.

[b17-cin-02-44] Li J, Orlandi R, White CN (2005). “Independent validation of candidate breast cancer serum biomarkers identified by mass spectrometry”. Clin Chem.

[b18-cin-02-44] Malone FD, Canick JA, Ball RH (2005). “First-trimester or second-trimester screening, or both, for Down’s syndrome”. N Engl J Med.

[b19-cin-02-44] Petricoin EF, Ardekani AM, Hitt BA (2002). “Use of proteomic patterns in serum to identify ovarian cancer”. Lancet.

[b20-cin-02-44] Postma R, Schröder FH (2005). “Screening for prostate cancer”. Eur J Cancer.

[b21-cin-02-44] Raaijmakers R Blijenberg BG Finlay JA 2004 “Prostate cancer detection in the prostate specific antigen range of 2.0 to 3.9 ng/ml: value of percent free prostate specific antigen on tumor detection and tumor aggressiveness” J Urol 1716 Pt 12245 9 15126795 10.1097/01.ju.0000127731.56103.50

[b22-cin-02-44] Schröder FH, Roobol-Bouts M, Vis AN (2001). “Prostate-specific antigen-based early detection of prostate cancer—-validation of screening without rectal examination”. Urology.

[b23-cin-02-44] Schröder FH, van der Maas P, Beemsterboer P (1998). “Evaluation of the digital rectal examination as a screening test for prostate cancer. Rotterdam section of the European Randomized Study of Screening for Prostate Cancer”. J Natl Cancer Inst.

[b24-cin-02-44] Skates SJ, Horick N, Yu Y (2004). “Preoperative sensitivity and specificity for early-stage ovarian cancer when combining cancer antigen CA-125II, CA 15-3, CA 72–4, and macrophage colony-stimulating factor using mixtures of multivariate normal distributions”. J Clin Oncol.

[b25-cin-02-44] Stephan C, Cammann H, Semjonow A (2002a). “Multicenter Evaluation of an Artificial Neural Network to Increase the Prostate Cancer Detection Rate and Reduce Unnecessary Biopsies”. Clin Chem.

[b26-cin-02-44] Stephan C, Jung K, Cammann H (2002b). “An artificial neural network considerably improves the diagnostic power of percent free prostate-specific antigen in prostate cancer diagnosis: results of a 5-year investigation”. Int J Cancer.

[b27-cin-02-44] Terry KL, Sluss PM, Skates SJ (2004). “Blood and urine markers for ovarian cancer: a comprehensive review”. Dis Markers.

[b28-cin-02-44] Vis AN, Hoedemaeker RF, Roobol M (2001). “Tumor characteristics in screening for prostate cancer with and without rectal examination as an initial screening test at low PSA (0.0–3.9 ng/ml)”. Prostate.

[b29-cin-02-44] Wald NJ, Cuckle HS, Sneddon J (1989). “Screening for Down syndrome”. Am J Hum Genet.

[b30-cin-02-44] Wald NJ, Densem JW, Smith D (1994a). “Four-marker serum screening for Down’s syndrome”. Prenat Diagn.

[b31-cin-02-44] Wald NJ, Hackshaw AK (1997). “Combining ultrasound and biochemistry in first-trimester screening for Down’s syndrome”. Prenat Diagn.

[b32-cin-02-44] Wald NJ, Kennard A, Hackshaw AK (1995). “First trimester serum screening for Down’s syndrome”. Prenat Diagn.

[b33-cin-02-44] Wald NJ, Kennard A, Smith D (1994b). “First trimester biochemical screening for Down’s syndrome”. Ann Med.

[b34-cin-02-44] Wald NJ, Rodeck C, Hackshaw AK (2003). “First and second trimester antenatal screening for Down’s syndrome: the results of the Serum, Urine and Ultrasound Screening Study (SURUSS)”. Health Technol Assess.

[b35-cin-02-44] Wallis C 2005 “The Down Dilemma.”Time Retrieved Dec. 15, 2005, from http://www.time.com/time/archive/preview/0,10987,1129545,00.html

[b36-cin-02-44] Woolas RP, Conaway MR, Xu F (1995). “Combinations of multiple serum markers are superior to individual assays for discriminating malignant from benign pelvic masses”. Gynecol Oncol.

[b37-cin-02-44] Zhang Z, Bast RC, Yu Y (2004). “Three biomarkers identified from serum proteomic analysis for the detection of early stage ovarian cancer”. Cancer Res.

[b38-cin-02-44] Zhang Z, Zhang H, Bast RC (2000). An application of artificial neural networks in ovarian cancer early detection.

